# Maternal age and psychological well-being in early pregnancy: a cross-sectional analysis

**DOI:** 10.3389/fpsyg.2026.1818113

**Published:** 2026-07-29

**Authors:** Juan Ran, Huanhuan Wang, Xiaoping Chen

**Affiliations:** Chengdu Wenjiang Maternal and Child Health Hospital, Chengdu, China

**Keywords:** anxiety, assisted reproductive technology, depression, maternal age, perceived stress, pregnancy

## Abstract

**Background:**

Psychological well-being in early pregnancy may differ by maternal age and by sociodemographic, economic, and reproductive circumstances. This study examined the associations of maternal age and other participant characteristics with screen-positive anxiety symptoms, screen-positive depressive symptoms, perceived stress, and composite psychological distress during the first trimester.

**Methods:**

A total of 1,282 women attending routine antenatal care at 10–12 weeks of gestation were included. Participants were classified into three maternal age categories: 18–29, 30–34, and ≥35 years. Psychological symptoms were assessed using the Edinburgh Postnatal Depression Scale, the Perceived Stress Scale, and the State–Trait Anxiety Inventory. Composite psychological distress was defined as meeting the prespecified threshold for screen-positive anxiety symptoms, screen-positive depressive symptoms, or high perceived stress. Associations were examined using multivariable logistic and linear regression models.

**Results:**

Mean state anxiety, trait anxiety, Edinburgh Postnatal Depression Scale, and perceived stress scores increased across maternal age categories. Screen-positive anxiety symptoms were present in 15.9, 23.2, and 33.0% of women aged 18–29, 30–34, and ≥35 years, respectively (*p* < 0.001); the corresponding prevalence of composite psychological distress was 28.9, 35.2, and 47.4% (*p* < 0.001). After adjustment, each one-category increase in maternal age was associated with higher odds of composite psychological distress (adjusted odds ratio [aOR] = 1.58, 95% CI 1.35–1.84; *p* < 0.001) and screen-positive anxiety symptoms (aOR = 1.76, 95% CI 1.48–2.10; *p* < 0.001), as well as a 0.20-point increase in perceived stress score (95% CI 0.11–0.29; *p* < 0.001). Conception through assisted reproductive technology and perceived financial strain were associated with higher odds of both binary outcomes. Perceived financial strain and work and career pressure were associated with higher perceived stress scores, whereas being currently married was associated with a lower score.

**Conclusion:**

Maternal age, conception through assisted reproductive technology, perceived financial strain, work and career pressure, and marital status were associated with psychological outcomes in early pregnancy. Prospective studies are needed to confirm these findings and identify feasible screening and support strategies for routine antenatal care.

## Background

Maternal psychological well-being is an important component of antenatal care. Systematic reviews indicate that depressive and anxiety symptoms are common during pregnancy, often co-occur, and vary across populations and assessment methods (Howard et al., 2014; Dennis et al., 2017; Yin et al., 2021; Falah-Hassani et al., 2017). Antenatal psychological symptoms have also been associated with preterm birth, low birth weight, impaired maternal functioning, and adverse developmental or relational outcomes for the child (Grote et al., 2010; Grigoriadis et al., 2013; Stein et al., 2014). Early pregnancy involves substantial physical change, uncertainty about pregnancy outcomes, and adjustment to new family and social roles, making it a relevant period for identifying women who may require further psychological assessment or support.

Psychological well-being during pregnancy is shaped by interacting clinical and social factors. Reviews have identified previous mental health problems, limited social support, stressful life events, relationship difficulties, and socioeconomic disadvantage as important correlates of antenatal anxiety and depression (Biaggi et al., 2016; Lancaster et al., 2010; Fisher et al., 2012). Maternal age may contribute through additional pathways. Women of advanced maternal age may have heightened perceptions of fertility, fetal, and obstetric risk, consistent with the greater frequency of adverse pregnancy outcomes reported in this group (Bayrampour et al., 2012; Lean et al., 2017; Laopaiboon et al., 2014; Carolan and Frankowska, 2011). Younger women may instead be more exposed to financial insecurity, limited preparation for parenthood, or unstable support. However, these experiences vary considerably, and maternal age alone is unlikely to capture psychological vulnerability.

Most research on maternal age has focused on obstetric and neonatal outcomes, whereas psychological status during the first trimester has received less attention. Pregnancy-specific stress, anxiety, and depressive symptoms overlap but remain conceptually and clinically distinct, and prevalence estimates differ according to the construct and instrument used (Falah-Hassani et al., 2017; Alderdice et al., 2012; Fawcett et al., 2019; von Elm et al., 2007). Studies that evaluate only one symptom domain or insufficiently account for socioeconomic and reproductive circumstances may therefore provide an incomplete picture.

We therefore conducted a cross-sectional study of women in early pregnancy. The primary objective was to examine the association between maternal age category and psychological well-being. The secondary objectives were to identify socioeconomic and pregnancy-related factors associated with screen-positive anxiety symptoms, perceived stress, and composite psychological distress.

## Materials and methods

### Study design and participants

This single-center cross-sectional study was conducted at Chengdu Wenjiang Maternal and Child Health Hospital. Women attending routine antenatal care at 10–12 weeks of gestation between January and December 2023 were assessed for eligibility. The study protocol was approved by the Ethics Committee of Chengdu Wenjiang Maternal and Child Health Hospital (approval no. WJFY2025LSLY-005). All study data were de-identified before analysis, and written informed consent was obtained from each participant.

Women were eligible if they had an ultrasound-confirmed pregnancy at 10–12 weeks of gestation and were able to provide informed consent. Exclusion criteria were a documented history of mental illness, multiple pregnancy, or chronic medical conditions. Maternal age categories were defined *a priori* as 18–29, 30–34, and ≥35 years. All included participants had complete data for the three psychological scales used to define the study outcomes. Screen-positive anxiety symptoms, screen-positive depressive symptoms, and high perceived stress were not mutually exclusive, because a participant could meet more than one screening threshold.

### Data collection and outcome definitions

Demographic and clinical information was collected using structured questionnaires and clinician-led interviews. Variables included maternal age, prepregnancy body mass index, gravidity, conception method, educational attainment, employment arrangement, monthly household income category, marital status, smoking history, history of alcohol use, self-reported readiness for childbearing, and self-reported stressors.

Psychological symptoms were assessed using the Edinburgh Postnatal Depression Scale (EPDS), which was developed for perinatal depressive symptom screening and has been evaluated during pregnancy and in Chinese women (Cox et al., 1987; Murray and Cox, 1990; Lee et al., 1998); the Perceived Stress Scale (PSS) (Cohen et al., 1983); and the State–Trait Anxiety Inventory (STAI) (Spielberger et al., 1983; Julian, 2011). An EPDS score ≥10 indicated screen-positive depressive symptoms. For this study, a PSS score >13 was classified as high perceived stress. Screen-positive anxiety symptoms were defined using prespecified thresholds of a state anxiety score ≥40, a trait anxiety score ≥35, or both. These screening classifications were intended to identify elevated symptoms and were not equivalent to clinical psychiatric diagnoses.

Monthly household income was categorized as low (<3,000 RMB), middle (3,000–10,000 RMB), or high (>10,000 RMB).

Composite psychological distress was defined as meeting the threshold for at least one of the following outcomes: screen-positive anxiety symptoms, screen-positive depressive symptoms, or high perceived stress. This study-defined composite outcome identified women with screening-level symptoms in one or more related domains.

### Statistical analysis

Analyses were performed using SPSS version 26 (IBM Corp., Armonk, NY, USA). Approximately normally distributed continuous variables were summarized as means and standard deviations, whereas skewed variables were summarized as medians and interquartile ranges. Categorical variables were summarized as counts and percentages. Differences among the three maternal age categories were assessed using one-way analysis of variance for approximately normally distributed continuous variables, the Kruskal–Wallis test for gravidity, and the chi-square test for categorical variables.

Multiple linear regression was used to examine factors associated with perceived stress scores. Binary logistic regression was used to examine factors associated with screen-positive anxiety symptoms and composite psychological distress. Maternal age category was entered as an ordinal variable with three levels: 18–29, 30–34, and ≥35 years. Linear regression results are reported as unstandardized coefficients (B), and logistic regression results are reported as aORs with 95% confidence intervals. All tests were two-sided, and *p* < 0.05 was considered statistically significant.

## Results

### Participant profile and psychological outcomes

**Figure 1:**
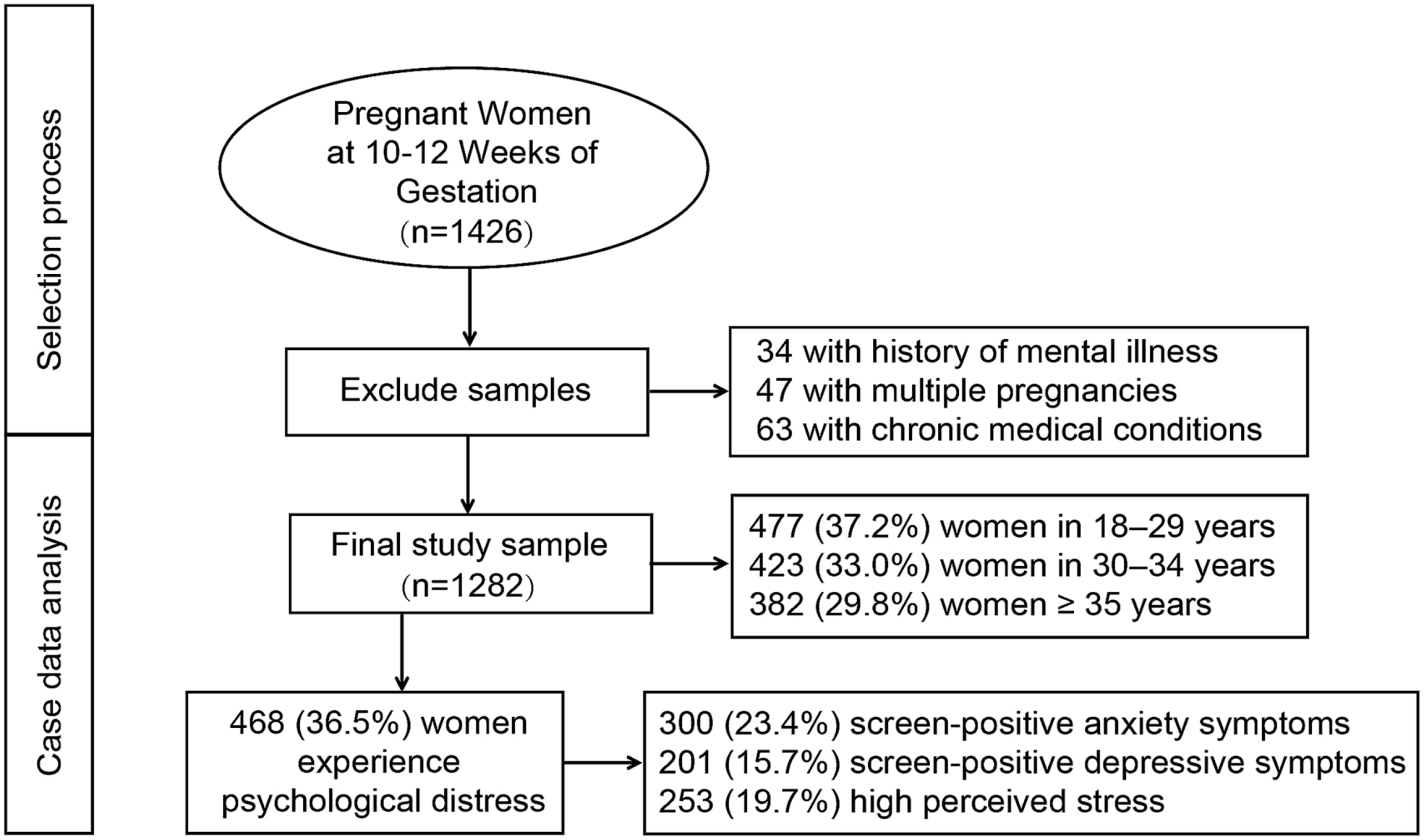
Participant selection, maternal age classification, and psychological screening outcomes for methodological checking.

Figure 1 summarizes participant selection, maternal age distribution, and psychological screening outcomes. A total of 1,426 women were screened. We excluded 34 women with a documented history of mental illness, 47 with multiple pregnancies, and 63 with chronic medical conditions, leaving 1,282 women in the analytic sample. Of these, 477 (37.2%) were aged 18–29 years, 423 (33.0%) were aged 30–34 years, and 382 (29.8%) were aged ≥35 years.

The mean maternal age was 31.41 ± 5.36 years, and the mean prepregnancy body mass index was 21.89 ± 2.68 kg/m^2^. A total of 103 women (8.0%) conceived through assisted reproductive technology (ART), and 776 (60.5%) reported being ready for childbearing (Table 1).

**Table 1:** Overall participant profile.

Characteristic	Overall sample
Sample size, *n*	1,282
Maternal age, years	31.41 ± 5.36
Maternal age category, years	
18–29	477 (37.2%)
30–34	423 (33.0%)
≥35	382 (29.8%)
Prepregnancy BMI, kg/m^2^	21.89 ± 2.68
Conception method	
Spontaneous conception	1,179 (92.0%)
ART conception	103 (8.0%)
Self-reported readiness for childbearing, yes	776 (60.5%)
Currently married	1,178 (91.9%)
Highest educational attainment	
Secondary school or below	149 (11.6%)
College or university education	865 (67.5%)
Postgraduate education	268 (20.9%)
Employment arrangement	
Not in paid employment	157 (12.2%)
Part-time employment or self-employment	298 (23.2%)
Full-time employment	827 (64.5%)
Monthly household income category	
Low income	186 (14.5%)
Middle income	929 (72.5%)
High income	167 (13.0%)
Psychological scale scores and screening outcomes	
State anxiety score	36.13 ± 2.99
Trait anxiety score	32.49 ± 2.25
Screen-positive anxiety symptoms	300 (23.4%)
EPDS score	7.73 ± 1.74
Screen-positive depressive symptoms	201 (15.7%)
Perceived stress score (PSS)	12.39 ± 1.33
High perceived stress	253 (19.7%)
Composite psychological distress	468 (36.5%)

ART, assisted reproductive technology; BMI, body mass index; EPDS, Edinburgh Postnatal Depression Scale; PSS, Perceived Stress Scale.

Composite psychological distress was identified in 468 women (36.5%), whereas 814 (63.5%) did not meet any component threshold. Screen-positive anxiety symptoms were identified in 300 women (23.4%), screen-positive depressive symptoms in 201 (15.7%), and high perceived stress in 253 (19.7%). The component outcomes were not mutually exclusive.

### Comparisons across maternal age categories

Participant characteristics differed significantly across maternal age categories with respect to ART conception, gravidity, smoking history, educational attainment, full-time employment, household income category, self-reported readiness for childbearing, and several self-reported stressors (Table 2). Prepregnancy body mass index, history of alcohol use, current marital status, pregnancy-related health concerns, and partner-related concerns did not differ significantly among the categories.

**Table 2:** Participant profile by maternal age category.

Characteristic	18–29 years (*n* = 477)	30–34 years (*n* = 423)	≥35 years (*n* = 382)	*p* value
Maternal age, years	25.64 ± 2.11	31.94 ± 1.05	38.02 ± 2.05	<0.001ᵃ
Prepregnancy BMI, kg/m^2^	21.80 ± 2.70	21.86 ± 2.56	22.02 ± 2.79	0.462ᵃ
Gravidity	1 (1)	2 (1)	2 (1)	0.001ᶜ
ART conception	15 (3.1%)	36 (8.5%)	52 (13.6%)	<0.001ᵇ
Smoking history	11 (2.3%)	7 (1.7%)	19 (5.0%)	0.012ᵇ
History of alcohol use	26 (5.5%)	12 (2.8%)	20 (5.2%)	0.123ᵇ
Currently married	440 (92.2%)	391 (92.4%)	347 (90.8%)	0.665ᵇ
Highest educational attainment				<0.001**ᵇ**
Secondary school or below	69 (14.5%)	34 (8.0%)	46 (12.0%)	
College or university education	329 (69.0%)	300 (70.9%)	236 (61.8%)	
Postgraduate education	79 (16.6%)	89 (21.0%)	100 (26.2%)	
Full-time employment	269 (56.4%)	279 (66.0%)	279 (73.0%)	<0.001ᵇ
Monthly household income category				<0.001**ᵇ**
Low income	61 (12.8%)	54 (12.8%)	71 (18.6%)	
Middle income	373 (78.2%)	310 (73.3%)	246 (64.4%)	
High income	43 (9.0%)	59 (13.9%)	65 (17.0%)	
Self-reported readiness for childbearing, yes	231 (48.4%)	261 (61.7%)	284 (74.3%)	<0.001ᵇ
Psychological scale scores and screening outcomes				
State anxiety score	35.41 ± 2.84	36.14 ± 2.98	37.01 ± 2.97	<0.001ᵃ
Trait anxiety score	31.95 ± 2.19	32.55 ± 2.19	33.08 ± 2.24	<0.001ᵃ
Screen-positive anxiety symptoms	76 (15.9%)	98 (23.2%)	126 (33.0%)	<0.001ᵇ
EPDS score	7.49 ± 1.70	7.72 ± 1.70	8.05 ± 1.78	<0.001ᵃ
Screen-positive depressive symptoms	59 (12.4%)	63 (14.9%)	79 (20.7%)	0.003ᵇ
Perceived stress score (PSS)	12.25 ± 1.28	12.33 ± 1.35	12.62 ± 1.34	<0.001ᵃ
High perceived stress	79 (16.6%)	81 (19.1%)	93 (24.3%)	0.016ᵇ
Composite psychological distress	138 (28.9%)	149 (35.2%)	181 (47.4%)	<0.001ᵇ
Self-reported stressors				
Work and career pressure	291 (61.0%)	296 (70.0%)	277 (72.5%)	<0.001ᵇ
Pregnancy-related health concerns	146 (30.6%)	132 (31.2%)	121 (31.7%)	0.944ᵇ
Parenting-related concerns	208 (43.6%)	132 (31.2%)	96 (25.1%)	<0.001ᵇ
Perceived financial strain	238 (49.9%)	159 (37.6%)	134 (35.1%)	<0.001ᵇ
Partner-related concerns	135 (28.3%)	130 (30.7%)	98 (25.7%)	0.279ᵇ

^a^Mean ± standard deviation; one-way analysis of variance. ^b^*n* (%); chi-square test. ^c^Median (interquartile range); Kruskal–Wallis test. ART, assisted reproductive technology; BMI, body mass index; EPDS, Edinburgh Postnatal Depression Scale; PSS, Perceived Stress Scale.

Mean state anxiety, trait anxiety, EPDS, and PSS scores increased across the three maternal age categories (all *p* < 0.001). The prevalence of screen-positive anxiety symptoms was 15.9, 23.2, and 33.0% among women aged 18–29, 30–34, and ≥35 years, respectively (*p* < 0.001). The corresponding prevalence of screen-positive depressive symptoms was 12.4, 14.9, and 20.7% (*p* = 0.003), and that of high perceived stress was 16.6, 19.1, and 24.3% (*p* = 0.016).

The prevalence of composite psychological distress was 28.9, 35.2, and 47.4% across the three maternal age categories, respectively (*p* < 0.001).

Self-reported stressors also varied by maternal age. Work and career pressure increased from 61.0% among women aged 18–29 years to 72.5% among those aged ≥35 years (*p* < 0.001). In contrast, perceived financial strain decreased from 49.9 to 35.1% (*p* < 0.001), and parenting-related concerns decreased from 43.6 to 25.1% (*p* < 0.001).

### Factors associated with composite psychological distress

In the multivariable logistic regression model, each one-category increase in maternal age was associated with higher odds of composite psychological distress (aOR = 1.58, 95% CI 1.35–1.84; *p* < 0.001). ART conception (aOR = 1.94, 95% CI 1.27–2.97; *p* = 0.002) and perceived financial strain (aOR = 2.77, 95% CI 2.17–3.54; *p* < 0.001) were also associated with higher odds of the composite outcome. No other covariate in the model was statistically significant (Table 3).

**Table 3:** Multivariable logistic regression for composite psychological distress.

Covariate	Adjusted OR	95% CI	*p* value
Maternal age category	1.58	(1.35–1.84)	<0.001
Prepregnancy BMI, kg/m^2^	1.03	(0.98–1.08)	0.199
Gravidity	1.01	(0.88–1.17)	0.851
ART conception	1.94	(1.27–2.97)	0.002
Currently married	0.79	(0.51–1.22)	0.283
Educational attainment	1.03	(0.83–1.27)	0.801
Full-time employment	1.01	(0.79–1.31)	0.913
Household income category	1.00	(0.80–1.26)	0.975
Work and career pressure	1.04	(0.80–1.34)	0.773
Perceived financial strain	2.77	(2.17–3.54)	<0.001

ART, assisted reproductive technology; BMI, body mass index; CI, confidence interval; OR, odds ratio.

### Factors associated with perceived stress scores and screen-positive anxiety symptoms

In the multiple linear regression model, each one-category increase in maternal age was associated with a 0.20-point increase in PSS score (*B* = 0.20, 95% CI 0.11–0.29; *p* < 0.001). Perceived financial strain (*B* = 0.65, 95% CI 0.51–0.80; *p* < 0.001) and work and career pressure (*B* = 0.33, 95% CI 0.18–0.48; *p* < 0.001) were associated with higher scores, whereas being currently married was associated with a 0.33-point lower score (*B* = −0.33, 95% CI − 0.58 to −0.07; *p* = 0.013) (Table 4).

**Table 4:** Multivariable linear regression for perceived stress scores.

Covariate	*B*	95% CI	*p* value
Maternal age category	0.20	(0.11, 0.29)	<0.001
Prepregnancy BMI, kg/m^2^	0.02	(−0.01, 0.05)	0.151
Gravidity	−0.02	(−0.10, 0.06)	0.622
ART conception	0.11	(−0.15, 0.37)	0.398
Currently married	−0.33	(−0.58, −0.07)	0.013
Educational attainment	−0.04	(−0.17, 0.08)	0.487
Full-time employment	0.07	(−0.07, 0.22)	0.324
Household income category	−0.02	(−0.16, 0.11)	0.746
Work and career pressure	0.33	(0.18, 0.48)	<0.001
Perceived financial strain	0.65	(0.51, 0.80)	<0.001

ART, assisted reproductive technology; BMI, body mass index; CI, confidence interval; PSS, Perceived Stress Scale.

In the adjusted logistic regression model for screen-positive anxiety symptoms, each one-category increase in maternal age was associated with higher odds of screen-positive anxiety symptoms (aOR = 1.76, 95% CI 1.48–2.10; *p* < 0.001). ART conception (aOR = 2.73, 95% CI 1.76–4.24; *p* < 0.001) and perceived financial strain (aOR = 3.22, 95% CI 2.43–4.27; *p* < 0.001) were also associated with higher odds of this outcome (Table 5).

**Table 5:** Multivariable logistic regression for screen-positive anxiety symptoms.

Covariate	Adjusted OR	95% CI	*p* value
Maternal age category	1.76	(1.48–2.10)	<0.001
Prepregnancy BMI, kg/m^2^	1.02	(0.97–1.08)	0.392
Gravidity	0.88	(0.75–1.04)	0.129
ART conception	2.73	(1.76–4.24)	<0.001
Currently married	1.02	(0.61–1.71)	0.938
Educational attainment	0.93	(0.73–1.19)	0.577
Full-time employment	0.98	(0.73–1.32)	0.910
Household income category	1.01	(0.78–1.31)	0.931
Work and career pressure	0.9	(0.67–1.21)	0.479
Perceived financial strain	3.22	(2.43–4.27)	<0.001

ART, assisted reproductive technology; BMI, body mass index; CI, confidence interval; OR, odds ratio.

### Factors associated with EPDS scores

In the multiple linear regression model, each one-category increase in maternal age was associated with a 0.25-point increase in EPDS score (*B* = 0.25, 95% CI 0.14–0.36; *p* < 0.001). ART conception (*B* = 0.38, 95% CI 0.07–0.69; *p* = 0.017), work and career pressure (*B* = 0.19, 95% CI 0.01–0.37; *p* = 0.038), and perceived financial strain (*B* = 0.72, 95% CI 0.55–0.89; *p* < 0.001) were associated with higher EPDS scores. Being currently married was associated with a lower EPDS score (*B* = −0.31, 95% CI − 0.61 to −0.01; *p* = 0.043). Prepregnancy BMI, gravidity, educational attainment, full-time employment, and household income category were not significantly associated with EPDS scores (Table 6).

**Table 6:** Multivariable linear regression for Edinburgh Postnatal Depression Scale scores.

Covariate	*B*	95% CI	*p* value
Maternal age category	0.25	(0.14, 0.36)	<0.001
Prepregnancy BMI, kg/m^2^	0.03	(−0.01, 0.07)	0.128
Gravidity	−0.01	(−0.11, 0.09)	0.842
ART conception	0.38	(0.07, 0.69)	0.017
Currently married	−0.31	(−0.61, −0.01)	0.043
Educational attainment	−0.05	(−0.20, 0.10)	0.515
Full-time employment	0.08	(−0.09, 0.25)	0.356
Household income category	−0.03	(−0.19, 0.13)	0.709
Work and career pressure	0.19	(0.01, 0.37)	0.038
Perceived financial strain	0.72	(0.55, 0.89)	<0.001

ART, assisted reproductive technology; BMI, body mass index; CI, confidence interval.

## Discussion

This study examined psychological well-being in early pregnancy in relation to maternal age, socioeconomic circumstances, and reproductive characteristics. Both continuous symptom scores and the proportions of women meeting screening thresholds increased across maternal age categories. The association with maternal age remained statistically significant after adjustment for demographic, socioeconomic, and reproductive covariates. However, screen-positive symptoms were observed in every age category, indicating that maternal age should be considered alongside other clinical and social factors rather than used as the sole basis for assessment.

Previous systematic reviews have shown that anxiety, depressive symptoms, and stress occur throughout pregnancy, although prevalence estimates vary according to the population, timing of assessment, construct, and instrument used (Howard et al., 2014; Dennis et al., 2017; Yin et al., 2021; Falah-Hassani et al., 2017; Fawcett et al., 2019). Emotional well-being may be affected by concerns about fetal health, pregnancy complications, employment, finances, family responsibilities, and the transition to parenthood. Such concerns are not confined to a single maternal age category.

Perceived financial strain was independently associated with all three modeled outcomes. Household income category, by contrast, was not independently associated with these outcomes, suggesting that objective income category and perceived financial strain are not interchangeable. Women with similar household incomes may differ in debt, housing costs, employment security, medical expenses, and family responsibilities. Antenatal assessment may therefore benefit from including simple questions about perceived financial strain rather than relying solely on income categories. Where appropriate, women experiencing substantial economic difficulties may be referred to available social welfare, financial counseling, or community support services.

ART conception was independently associated with composite psychological distress and screen-positive anxiety symptoms, consistent with previous systematic reviews of psychological adjustment after assisted conception (Verhaak et al., 2007; Hammarberg et al., 2008). Some women who conceive through ART have experienced infertility, repeated procedures, pregnancy loss, or substantial treatment costs. Pregnancy after ART may therefore remain emotionally demanding even after conception. This finding does not imply that ART conception itself constitutes a psychological disorder; rather, reproductive history may help clinicians identify women who could benefit from additional information, reassurance, or follow-up.

The findings suggest that psychological assessment should not be restricted to women selected solely on the basis of maternal age, household income category, or conception method. Current clinical guidance supports the use of validated instruments during pregnancy when screening is embedded within systems that can provide assessment, treatment, and follow-up (American College of Obstetricians and Gynecologists, 2023a; American College of Obstetricians and Gynecologists, 2023b). Where clinical resources and referral pathways are available, brief screening may be incorporated into the first antenatal or pregnancy registration visit and conducted in a private, supportive setting. Women should be informed that its purpose is to understand their well-being and identify possible needs for further support.

A positive screening result should prompt further assessment rather than be treated as a diagnosis. Follow-up assessment may consider symptom severity, duration, functional impairment, sleep, social support, previous mental health history, and safety concerns. Evidence reviews similarly emphasize that the benefits of screening depend on accurate assessment and access to effective care rather than screening in isolation (American College of Obstetricians and Gynecologists, 2023a; American College of Obstetricians and Gynecologists, 2023b; O'Connor et al., 2016). Women with mild or short-term distress may benefit from information, brief counseling, stress-management guidance, peer support, and planned reassessment. Persistent or more severe symptoms may require structured psychological treatment or specialist evaluation.

Psychological care during early pregnancy should also include prevention and health promotion for women who do not meet screening thresholds. Counseling interventions can reduce the likelihood of perinatal depression among women at increased risk, and recent perinatal guidance supports individualized preventive and therapeutic planning (Curry et al., 2019; Vigod et al., 2025). Antenatal education may address common emotional changes, practical approaches to stress management, warning signs that warrant professional attention, and available sources of support. Such information may help women and their families recognize difficulties earlier and reduce hesitation in seeking care.

This study has several strengths. It included a relatively large sample of women in early pregnancy and used validated instruments to assess anxiety, depressive symptoms, and perceived stress. Both composite and domain-specific screening outcomes were examined, with adjustment for relevant demographic, socioeconomic, and reproductive factors. The focus on the first trimester also provides clinically relevant evidence for early antenatal assessment.

Several limitations should be acknowledged. The cross-sectional design precludes causal inference, and the single-center setting may limit generalizability. Psychological outcomes were based on self-reported screening scales rather than diagnostic interviews. Some potentially relevant factors, including previous pregnancy loss, family psychiatric history, relationship quality, and social support, were not assessed in detail, and residual confounding is possible. In addition, because participants were recruited from a hospital-based antenatal population rather than through population-based sampling, the sample may not fully represent all pregnant women in the local community. Future multicenter and longitudinal studies should examine changes in psychological symptoms throughout pregnancy and assess how clinical, family, workplace, and community support influence maternal well-being. Intervention studies are also needed to determine which screening and support strategies are feasible and beneficial in routine antenatal settings.

## Conclusion

Older maternal age category, ART conception, and perceived financial strain were associated with higher odds of selected psychological screening outcomes in early pregnancy. Work and career pressure was associated with higher perceived stress, whereas being currently married was associated with lower perceived stress. Early antenatal assessment should consider both social and reproductive circumstances and, consistent with current evidence and guidance, should be linked to diagnostic assessment, appropriate intervention, and follow-up rather than implemented as screening alone. Prospective studies are needed to confirm these findings and identify feasible screening and support strategies for routine antenatal care.

## Data Availability

The original contributions presented in the study are included in the article/supplementary material, further inquiries can be directed to the corresponding author.
